# Eyewitness Memory for Person Identification: Predicting Mugbook Recognition Accuracy According to Person Description Abilities and Subjective Confidence of Witnesses

**DOI:** 10.3389/fpsyg.2021.675956

**Published:** 2021-08-13

**Authors:** Alexander Handler, Sascha Frühholz

**Affiliations:** ^1^Cognitive and Affective Neuroscience Unit, University of Zurich, Zurich, Switzerland; ^2^Neuroscience Center Zurich, ETH Zürich, University of Zurich, Zurich, Switzerland; ^3^Department of Psychology, University of Oslo, Oslo, Norway

**Keywords:** mugbook, eyewitness identification, person description, person recognition, memory

## Abstract

Mugbook searches are conducted in case a suspect is not known and to assess if a previously convicted person might be recognized as a potential culprit. The goal of the two experiments reported here was to analyze if prior statements and information about the suspect can aid in the evaluation if such a mugbook search is subsequently advised or not. In experiment 1, memory accuracy for person descriptors was tested in order to analyze, which attributes could be chosen to down-scale the mugbook prior to testing. Results showed that age was the most accurate descriptor, followed by ethnicity and height. At the same time self-assessed low subjective accuracy of culprit descriptions by the witness seemed to be divergent to the objective actual performance accuracy. In experiment 2, a mugbook search was conducted after participants viewed a video of a staged crime and gave a description of the culprit. Results showed that accuracy in mugbook searches correlated positively with the total number of person descriptors given by the witness as well as with witness’ description of external facial features. Predictive confidence (i.e., subjective rating of own performance in the subsequent mugbook search), however did not show any relation to the identification accuracy in the actual mugbook search. These results highlight the notion that mugbooks should not be conducted according to the subjective estimation of the witness’ performance but more according to the actual statements and descriptions that the witness can give about the culprit.

## Introduction

Eyewitness testimonies are a central part of many investigative processes on crimes and strongly depend on the memory, culprit description, and person recognition abilities of witnesses. These investigative procedures for eyewitness identification of crime culprits can be divided into different types depending on how the identification process is conducted. In case of a known culprit, either a so-called “show-up” or a “line-up” procedure can be conducted. Show-up procedures consist of a witness trying to identify a culprit in person, for example, at a police station or in court. In a line-up procedure, a witness is looking through a series of pictures and is trying to identify the picture of the culprit. If there is no known culprit, a third visual identification technique can be conducted. In this so-called “mugbook search” the witness is asked to look through a mugbook (i.e., collection of mugshots) to see if they recognize anyone as the potential culprit. If the culprit has been arrested before, a picture might be on file, and the witness will possibly recognize him/her as the culprit. Mugbook searches are usually conducted when law enforcement has not yet narrowed in on a possible suspect.

While there has been a lot of research done in line-up and show-up procedures, research is scarcely done on the psychological and cognitive processes involved in mugbook searches (Lindsay et al., 2017). While there are similarities between the different procedures, there are also crucial differences so that one could question the generalization from one procedure to the other (McAllister et al., 2008). Research on mugbook searches is usually conducted to gain insights in how accurately people perform when they have to review a large sample size of person pictures (Blunt and McAllister, 2009; Kalmet, 2016) or how to evaluate differences in line-up procedures and mugbook searches (Stewart and McAllister, 2001; McAllister et al., 2008). It is generally assumed that mugbook searches can be conducted easily if no culprit is readily available, but it seems reasonable to take some caution before conducting a mugbook search. Mugbook searches should be limited to situations where a positive identification seems likely, and the performative quality and success of mugbook searches depends on critical features as we outline in the following sections.

### Variables Influencing Identification Accuracy

Research on eyewitness identification accuracies of potential culprits generally distinguishes between system and estimator variables that influence the identification accuracy of witnesses (Wells and Lindsay, 1985; Wells et al., 2006).

System variables are those that can be directly controlled by the justice system and can be controlled by law enforcement units during the identification process (Wells et al., 2006). These system variables include the instructions given prior to the identification task (e.g., Malpass and Devine, 1981), the procedure of how the pictures are presented (grouped vs. sequential; McAllister et al., 2008), the number of pictures presented, and the order the pictures are presented (e.g., Kalmet, 2016).

Estimator variables on the other hand, can be controlled and manipulated in scientific research, however their effect on eyewitness recognition can only be estimated by police forces in real criminal cases (Wells et al., 2006). Estimator variables usually affect the witness as the crime takes place or at least before the mugbook search is conducted and include the ethnicity of the culprit and witness (e.g., Davis and Loftus, 2018), the focus of attention (Davis and Loftus, 2018), the experience of stress (Deffenbacher et al., 2004), the use of a weapon during the crime (Fawcett et al., 2013), the exposure duration and possible disguises of the culprit (Cutler et al., 1987), and the delay between crime and identification (Shapiro and Penrod, 1986).

### Description Accuracy of Culprit Features

Person descriptions given by eyewitnesses have been largely neglected for a long time, with only scarce scientific knowledge available until now (Fahsing et al., 2004). Person descriptions given by victims or witnesses frequently apply to many potential persons and tend to be rather non-distinctive (Meissner et al., 2007). During free recall to describe an unfamiliar person, witnesses usually provide information about gender, age, height, build, race, hair color, and clothing (Kuehn, 1974; Lindsay et al., 1994a; Voelkle et al., 2012). However, specific features of the face, for example, are rarely mentioned (Lindsay et al., 1994a). While some descriptors tend to be useful to describe an unknown person, others tend to be problematic, especially in the identification process. For example, clothing or references to the hair are likely to be of little help as they can readily be altered by a culprit (Meissner et al., 2007).

Person descriptors can therefore be divided into permanent and temporary characteristics (Van Koppen and Lochun, 1997), and facial descriptors can be additionally divided into external features (e.g., head shape, hair, ears) or internal features (e.g., eye color, nose, mouth) (Ellis, 1992). While identifications of familiar persons rely more on internal features, external features seem to be more important to recognize a stranger, such as a culprit in a mugbook search (Davis and Loftus, 2018). There are also additional variables that influence the witness’ memory for culprits. Memory information about a culprit is always partly combined with a prior estimate of a person’s characteristics. These estimates related to certain person characteristics seem to be affected by the knowledge about central person categories. Specifically, estimates tend to be adjusted to typical (central) category values that are associated with certain person categories (Twedt et al., 2015).

Previous work has shown that witnesses, in real-life cases, tend to be fairly precise when describing a culprit (Fahsing et al., 2004), however, this work should be treated with caution. First, one rarely knows for sure who truly committed the crime. Second, the person depicted in the police file and the person described by the witness might not be the same. And third, accuracy scoring is possible only for offender attributes that are known to the police, and certain information may simply not be verifiable (Fahsing et al., 2004). Additionally, it is nearly impossible to isolate variables, as they might be correlated with other variables of the crime, or they might not even have been measured (Wells and Penrod, 2011). The first experiment of this paper will therefore investigate in an experimental setting how accurate person descriptions actually tend to be if the potential culprit was seen only for a short time period.

### Description and Identification

The level of accuracy of person descriptions by witnesses may be an important factor that influences the arrangement for a mugbook, especially since the verbal description of a culprit might be associated with the later identification of the culprit. Current research assumes a rather small relationship between a prior description and a subsequent identification at best (Meissner et al., 2007). This might be because description and identification draw on different types of knowledge. While descriptions depend on participants’ memory for distinctive features, identifications depend on their non-verbal knowledge of the face in its entirety (Farah et al., 1998). An important scientific question is therefore to analyze the descriptions and investigate how the processing of providing a description might cause a better or worse identification in a subsequent mugbook search.

An important factor affecting eyewitness identification is when a statement about the culprit was given prior to the identification process. This effect is called “verbal overshadowing.” Verbal overshadowing describes the effect that eyewitness identifications tend to be less accurate when people had to give a verbal description of the culprit compared to non-verbal descriptions (Mickes, 2016). This finding might be counterintuitive as describing a face might represent a verbal rehearsal, which usually should enhance memory performance. However, verbal person descriptions may encourage a witness to focus upon expressible features of a face, which may not be helpful when the face has to be recognized in a visual identification task due to the different cognitive processes required in both tasks (Meissner et al., 2007).

In addition to verbal overshadowing, Gabbert et al. (2009) argued that if a witness recalled only an incomplete subset of information from memory in the description phase, this would impair the ability to subsequently recall the remaining not previously recalled items of information that are relevant for the identification process. This would lead to the conclusion that if a witness does not mention information about the culprit in verbal statements, he/she would not be looking for those details in a subsequent identification task.

These findings and conclusions led to the main question of the studies presented here. We aimed to analyze factors and especially estimator variables that influence the success of the mugbook search (i.e., identification of the culprit) based on the description a witness gives about the culprit prior to mugbook search. We tested the following hypothesis; first, we expected that the quality of the statements given by the witness during the culprit description phase will be positively correlated with the ability to correctly identify a subject in a following mugbook search. Second, we expected that witnesses will describe significantly more external than internal facial features during the person description phase. And third, we expected a positive correlation between the number of correctly remembered external facial features and the identification accuracy in the mugbook search.

### Witnesses’ Subjective Confidence and Identification Accuracy

Our study also included a fourth hypothesis that concerned more subjective factors that influence the preparatory and performative process of mugbook searches. An important additional feature during the description and identification phase is the subjective confidence of the witness in his/her statements and decisions. Eyewitness reports tend to be less objective than other forms of evidence, however, they are often perceived as more convincing than other types of evidence, especially if such reports are expressed with high levels of confidence (Nash et al., 2015). The true effect of confidence on description and identification accuracy, in contrast, was not as well documented for a long time.

Kassin (1984) reported that the correlation between accuracy and confidence was not significant for physical descriptions. Concerning identification accuracy, the relationship to confidence varies greatly depending on many other factors, such as memory strength, the fact that people identified a culprit or rejected the line-up, and the similarity of the line-up (Wells et al., 2006). In recent years, eyewitness experts have started to recognize that the confidence of the witness at the time of first identification strongly predicts accuracy (Wixted and Wells, 2017). However, the relation between accuracy and confidence has to be considered with caution as confidence can change over time and, even though memory strength fades, confidence can become stronger (Davis and Loftus, 2018).

Confidence can be assessed before conducting the identification task (e.g., “How certain are you that you will recognize the culprit in a mugbook search?”) or after conducting the identification task (e.g., “How certain are you that you recognized the culprit/that the culprit was not in the mugbook”). These two types of certainty can be divided into predictive confidence and postdictive confidence (Nguyen et al., 2018). A number of papers have shown that postdictive confidence is associated with accuracy especially for witnesses who chose someone as being the culprit compared to non-choosers (Wixted and Wells, 2017; Davis and Loftus, 2018; Dobolyi and Dodson, 2018; Nguyen et al., 2018).

Concerning predictive confidence, the literature is not so optimistic, and previous research usually found that if witnesses had to predict their own performance on an upcoming identification task, they often overestimated their accuracy in the identification task (Cowan et al., 2014). Furthermore, the correlation between predictive confidence and accuracy was often non-existent or small at best (Cutler and Penrod, 1989; Sauerland and Sporer, 2009; Valentine and Mesout, 2009). Therefore, an additional fourth hypothesis that was investigated here is that predictive confidence in witnesses will show a positive correlation with actual performance in mugbook searches.

To assess the aforementioned questions and four hypotheses, we conducted two independent experiments. Experiment 1 was conducted to analyze how accurately certain person descriptions are given by witnesses in the description phase and which of these description features could be used to select mugshots for a subsequent mugbook search. Experiment 2 simulated an actual mugbook search to obtain insight into the relationship between person descriptions and identification accuracy in witnesses. Participants in our experiments where therefore asked to describe a potential culprit that they have seen in a short encounter or in a video of a simulated crime. Participants were later asked to identify the culprit in a mugbook search, especially in experiment 2. Both experiments were conducted to investigate the entire process of a mugbook search, including the culprit description phase and the mugbook search phase.

## Experiment 1–Eyewitness Memory for Culprit Features

A typical mugbook search usually includes two separate phases. In a first preparatory phase, the witness gives a description of the potential culprit and is asked by criminal investigators to provide all details that the witness can remember about the potential culprit. This preparatory phase of the culprit description serves the selection of mugshots for the subsequent mugbook search as the identification phase. In experiment 1, we aimed to experimentally simulate this first preparatory phase, while witnesses were asked to give person descriptions of a shortly encountered culprit. Short and rather incidental encounters are very typical for real-life crime scenes.

### Methods

#### Participants as “Witnesses”

A total of 86 participants were tested. The sample consisted of 20 men (23.3%), 65 women (75.6%) and one person that identifies as a non-binary person. The average age was 22.41 years and ranged from 17 to 36 years (*M* = 22.41, SD = 4.11). 29 participants (34%) are in high school, 39 (45%) are psychology students at the University of Zurich (Switzerland), and 18 (21%) are students at the Police Academy of Zurich (Switzerland).

#### Memorized Person as Simulated “Culprits”

To assess how accurately different descriptive attributes of potential culprits can be described, five different male persons were presented and introduced as “culprits,” however, without being involved in an actual police case or a simulated crime. The people were chosen to reflect “typical” attributes of a culprit. The exact attributes of the culprits can be found in Table 1. Three culprits were of age 27 years, one was 30 years, and one 31 years. The smallest culprit was 163 cm tall and the tallest 186 cm tall. The others measured 175, 178, and 180 cm. All of the culprits were western European, three of them had brown hair, and two had blond hair. Two culprits had blue eyes, two had green eyes and one had brown eyes. None of them had any visible tattoos, and only one had a visible piercing being an earring on the left side. The culprit would always wear a black T-shirt, and in every context only one culprit was presented.

**TABLE 1:** Description of culprits in Experiment 1.

**Descriptors**	**Culprit 1**	**Culprit 2**	**Culprit 3**	**Culprit 4**	**Culprit 5**
Age	30	27	27	31	27
Height	163	180	178	186	175
Ethnicity	Western European	Western European	Western European	Western European	Western European
Hair color	Brown	Brown	Blond	Blond	Brown
Eye color	Green	Brown	Blue	Blue	Green
Tattoo	–	–	–	–	–
Piercing	–	–	Left ear	–	–

*Experiment 1, Age was measured in years. Height noted in centimeters. All culprits wore a black T-shirt.*

#### Questionnaire

The questionnaire handed to the participants was an adapted version of the questionnaire used by the city police in Zurich (Switzerland) to prepare for the arrangement and selection of the pictures presented in a subsequent mugbook search. The questionnaire was presented in German, so the participants all had to be German-speaking. Participants were first asked how good their memory of the culprit was, which they could answer on a five-point Likert scale ranging from “very bad” (1) to “very good” (5). Participants were then asked to give written information about age, height, ethnicity, hair color, eye color, tattoos and piercings. Except for ethnicity, all questions were open questions with the possibility to answer “I do not recall” in every question.

#### Experimental Procedure

The culprit would show up at the beginning of common teaching sessions at the respective institutions together with the examiner. Both the examiner and the culprit would be introduced as examiners of a short memory study which would be conducted in class. The culprit would hand out a paper turned upside down in a sheet protector directly to every class attendee, which would say: “Remember the examiner with the black T-shirt.” As soon as the culprit was next to the examiner again, the participants were asked to turn around the paper and do the task mentioned on the paper. Five seconds after giving this instruction, the culprit would leave the room, so the participants only viewed the culprit for a short amount of time while knowing that they had to remember him leading to an identical memorizing time for every session. However, it should be noted that the amount of time participants observed the culprit while he was handing out the papers (before they were instructed to remember him) may have varied and was not directly controlled by the experiment.

The participants were then asked to retrieve features of the culprit, which they would generally use to describe him to another person, and were asked not to talk about him with the other class attendees. Participants then had their classroom sessions as scheduled. At the end of their session, the examiner entered the room again, but this time without the culprit, and handed out the questionnaire to the participants.

The experiment was conducted in this way to resemble a possible real-life theft without having to stage a theft. As in a theft, the culprit might be seen up close, but the victim often only realizes the theft after the perpetrator has left or is leaving and while the victim is looking e.g., for his or her wallet. The creation of a similar situation was attempted, with the participants being able to view the culprit in proximity while distributing the sheet protector, yet they only learned that they had to remember the culprit once he is not as close anymore.

#### Data Analysis

To analyze the questionnaire data, the information given on ethnicity, hair color, and eye color was rated as correct, incorrect, or missing (if the person stated, “do not recall”). In the open questions, answers would count as correct if at least part of the answers were correct. For example, if eye color was stated as blue-green and the true color was blue, this would be correct. If only dark hair was stated instead of brown, this would be counted as a missing value. The reason, therefore, is that when selecting pictures to conduct a mugbook search later and all culprits with blue or green eyes are included, the picture would be included yet dark hair would not be precise enough to include or exclude pictures. Age and height were not rated as correct or incorrect, as it is hard to determine what estimation can still be counted as correct and what not (Meissner et al., 2007). In these categories, accuracy was measured as the difference between estimated and true value. If a person did not estimate a certain age but a range instead (e.g., 20–30 years or 175–180 cm) the mean of the range would be used as an answer (e.g., 25 years or 177.5 cm). Data were collapsed over all the culprits collectively as the design of the experiment would not allow identifying differences between culprits or classes, as each culprit was present in only one class.

### Results

#### Age Estimation

According to the age of the potential culprit, the mean difference of estimate and true age was 2.26 years younger (SD = 2.71), ranging from −9 years to +4 years, which is considered a significant difference to zero according to a Wilcoxon Signed-rank test (*z* = −6.144, *p* < 0.001). When the distribution of these estimates are examined, 16.5% (*n* = 14) reported an age difference that exceeded 5 years, and 40% (*n* = 34) had differences of more than 2.5 years.

#### Height Estimation

**FIGURE 1:**
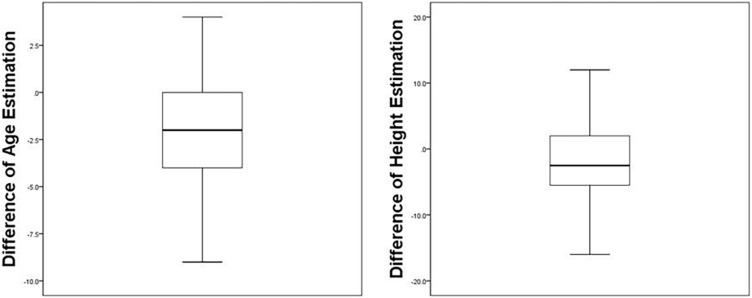
The left boxplot shows the distribution of the difference of estimated age and true age of the culprit. Numbers represent the differences in years. The right boxplot shows the distribution of the difference of estimated height and true height of the culprit. Numbers represent the differences in centimeters.

Differences from estimated to true height ranged from −16 cm to +12 cm (*M* = −1.87 cm, SD = 5.32) which represents a significant difference to zero according to a Wilcoxon Signed-rank test (*z* = −3.271, *p* = 0.001). Distributions of the height estimations show that 33% (*n* = 28) estimated the culprit to be more than 5 cm taller or smaller. Plots of the estimates can be found in Figure 1.

#### Ethnicity Estimation

Assumptions of the ethnicity of the culprit were correct for 65 participants (75.6%), with four participants (4.7%) not being able to recall the ethnicity and 17 participants (19.8%) reporting an incorrect ethnicity of the culprit.

#### Hair Color Estimation

Hair color was remembered correctly by 44 participants (51.2%) and incorrectly by 31 participants (36%), with eleven participants (12.8%) not being able to recall the hair color. Most of the participants (*n* = 40, 46.5%) reported that they did not see or remember the eye color. 20 participants (23.3%) were able to report the correct eye color, while 26 (30.2%) reported an incorrect color.

#### Subjective and Objective Memory Estimate

To determine the relationship of how well people thought they could remember the culprit and their true features, the relationship between the estimation of the own memory accuracy and the actual person descriptor accuracy was measured. Overall, 16.3% of participants (*n* = 14) rated their memory on the lower half of the scale, while 39.6% (*n* = 34) of the participants were on the upper half of the scale, meaning they were rather confident in their memory and 44.2% of participants (*n* = 38) rated their memory as neither good nor bad. For age and height, a simple two-tailed Spearman-Rank-correlation was calculated as age (*D* = 0.138, df = 85, *p* < 0.001) and height (*D* = 0.098, df = 85, *p* = 0.042) both did not show normal distribution according to the Kolmogorov-Smirnov test. Neither the correlation of memory estimation of age and actual accuracy of age (rs = −0.003, *p* = 0.489) nor the correlation of memory estimation of height and actual accuracy of height (rs = −0.053, *p* = 0.314) showed a significant correlation. Similar results were obtained for estimations of ethnicity, hair color, and eye color, which were analyzed with the Chi-Squared test to see if the results in accuracy depended on the memory. The tests showed that memory strength did not affect accuracy of ethnicity (χ^2^ = 7.370, df = 4, *p* = 0.118), hair color (χ^2^ = 4.629, df = 4, *p* = 0.329), and eye color (χ^2^ = 2.406, df = 3, *p* = 0.493).

### Discussion

Results from experiment 1 support the findings by Kuehn (1974) that witnesses would provide a good description about more general culprit features but were less accurate in specific features such as hair or eye color. Also, our results are similar to the numbers found by Fahsing et al. (2004), which are that general attributes (height, ethnicity, and age) showed better accuracy compared to hair and eye color, which were not remembered very accurately.

The question nevertheless remains whether a good general description and descriptive features provide a valid basis for decisions about which culprit pictures are included in the subsequent mugbook search and which not. If age of the culprit (±5 years) is considered as the only criterion, 83.5% of the subjects would create a mugbook that included the culprit. If only height of the culprit (±5 cm) is considered, then 67% of the mugbooks would feature the culprit. And if only ethnicity is considered, then 75.6% of the mugbooks would include the culprit.

However, especially in large cities or populations, where many thousands or up to a million mug-shots might be stored (Lindsay et al., 2017), the use of only one attribute to downscale the picture array might be insufficient. If height, age, and ethnicity are all jointly considered to reduce the number of persons potentially included in the final mugbook arrangement, then only 43 participants (50%) in our study would have created a mugbook that included the culprit. These results seem rather daunting. It seems necessary to minimize the mugbook size, yet the use of only three attributes leads to a 50% chance of the culprit not even being present in the mugbook and therefore making it impossible to correctly identify the culprit. It appears to be ideal to use a matching approach with caution and consider a sequencing approach when attributes are considered with a high error rate.

Interestingly, the assessment of the participants’ subjective memory abilities of the culprit and culprit features did not show any relation with the true accuracy of person descriptors, which supports the findings of Kassin (1984). A possible explanation for this might be that people are simply not good at estimating their own memory capability, yet it is also possible that these differences were found because people were accurate in their estimation of their memory, however, the estimations were wrong from the beginning. The latter could indicate that participants were overall inaccurate in making judgments about culprit traits directly, which would indices a general cognitive difficulty in judging person traits rather than a subsequent memory problem. Future studies thus might be interested in finding out if people were able to encode attributes in the first place and forgot them subsequently or if they were not able to encode them correctly meaning it would not be a problem of their own memory and therefore the judgment of the own memory accuracy might be right but the attribute all together was wrong.

## Experiment 2–Variables Influencing Identification Accuracy in a Mugbook Search

While experiment 1 aimed to simulate the preparatory phase of a mugbook search in an experimental setting, experiment 2 aimed to experimentally simulate the mugbook search itself as the second phase of a mugbook investigation. In experiment 2, witnesses encountered a staged crime and were subsequently asked to identify the potential culprit in an experimentally controlled mugbook search.

### Method

#### Participants as “Witnesses”

The experiment included an independent sample of 120 participants. Two participants did not understand the questionnaire correctly and had to be excluded, as they incorrectly answered the questions about a simulated crime they had seen prior to participating in the experiment. The remaining 118 participants consisted of 82 female (69.5%) and 36 male (30.5%) participants with an age range of 19–75 years (*M* = 30.23 years, SD = 12.08). 98 participants (83.1%) were from Switzerland. Additionally, there were 11 participants (9.3%) from Germany, four participants (3.4%) from Austria, and five participants had a background of another country. All participants were fluent in German as the whole experiment was conducted in German. Regarding the highest level of education, two participants were junior high school graduates (1.7%), 11 technical college graduates, 42 senior high school graduates (35.6%), 21 University of Applied Science student (17.8%), and 42 University students (35.6%). 63 participants were currently studying (53.4%). 78 participants (66.1%) did not have prior experience with burglary, while 17 (14.4%) have experienced a theft as victim and 23 (19.5%) as a witness. Nine (7.6%) people had previous experience with eyewitness identification tasks, either in a research or a legal setting.

#### Video of Simulated Crimes and Culprits

Participants were shown one of two videos depicting a non-violent theft of a handbag. Both movies were identical except for the perpetrator. The movie lasted 3 m 15 s with the theft taking place in the final seconds of the movie and the perpetrator being seen for 45 s overall with a clear view of his face for approximately half the time. The video was made with cuts in a movie like manner and the culprit was seen from different distances (varying from 1 to 20 m approximately). The only people seen in the movie were the victim, a witness, and the perpetrator. Two culprits were chosen to minimize the chance that the results are affected by distinct features of one specific culprit (Badham et al., 2013).

#### Mugbook Procedure

A mugbook was created, including a total of 300 pictures. Most faces depicted in the mugbook were Caucasian males, however to increase the size of the mugbook, a small amount of pictures depicted Arabic or Latin faces. To create a mugbook of 300 pictures, 299 were selected from the following face databases: FACES Database (Ebner et al., 2010), CVL Database (Peer et al., 2014), Chicago Face Database (Ma et al., 2015), MIT CBCL face recognition Database (Weyrauch et al., 2004), Radboud Face Database (Langner et al., 2010), and the Siblings Database (Vieira et al., 2014). All pictures used were in color, depicting males with neutral emotional expression in frontal view. All pictures were edited to have a white-gray background and cropped to have the head sizes matched.

The picture of the culprit was added to the other 299 pictures creating two separate mugbooks, each consisting of 300 pictures. The picture of the culprit matched the other pictures in the mugbook search. Instead of creating an extra culprit absent mugbook, the mugbook containing the picture of the culprit not seen in the movie was used when an absent mugbook was tested. The order of the pictures, including the culprit was randomized. The size of the pictures was ∼7 × 5.5° of the visual angle each.

#### Questionnaire

The questionnaire contained short demographic questions, a slightly adapted version of the Self-Administered Interview (SAI) (Gabbert et al., 2009) in German, and additional questions about the crime. The SAI was developed on the basis of the Cognitive Interview Technique (Gabbert et al., 2009). The “Cognitive Interview” is an investigative interviewing technique to enhance memory performance based on various principles of memory retrieval (Geiselman et al., 1985; Fisher and Geiselman, 1992). The SAI offers a tool for witnesses to recall their memories of an incident by themselves, following specific instructions and questions. The SAI supports people to give a complete description of everything the witness has seen in the crime and follows up with more specific questions about the perpetrator and other details of the crime (Gabbert et al., 2009). The SAI was used to support the witness to provide as much information as possible without the necessity of an interaction between subject and examiner, which could affect the results.

After the SAI questionnaire, predictive confidence for the later mugbook task was assessed with the question, “How certain are you that you would correctly identify the culprit in the mugbook task?” Answers were given on a six-point Likert Scale from “very improbable” (1) to “very probable” (6).

#### Experimental Procedure

The 120 participants were randomly assigned in a 2 (culprit A vs. culprit B) × 2 (culprit present vs. absent) design. As mugbook searches tend to show less correct identifications due to the large number of pictures seen (Meissner et al., 2007) the first 60 participants were randomly assigned between the two culprits, and they all received a culprit present mugbook to increase the possible number of correct identifications and thus to enhance the chance of more recognized targets as the goal was to know if recognition of targets can be predicted. The second 60 participants were randomly assigned to the different conditions in the aforementioned 2.

Participants were told they would participate in a study about daily activities. They were tested individually, and the session lasted approximately 1 h. Participants viewed a short movie and were told to watch the movie attentively without knowing a crime was about to happen. After the movie, they were instructed to memorize the theft they had witnessed and were told that they would be questioned about what happened later. They were given a 10 min distractor task (i.e., an easy crossword puzzle and two paper-pencil labyrinths) between the end of the video and the subsequent memory retrieval phase to decrease the possibility of the culprit just being remembered in the working memory.

Following the distractor task, participants were asked demographic questions about the culprit followed by the SAI and some additional questions to the crime. In the end, they were asked how likely they were to recognize the perpetrator in a following mugbook search if he was present. Subsequently, they completed the mugbook search. The search included either the culprit present or culprit absent mugbook. Pictures were presented in a grouped procedure resulting in 15 pictures per page, and 20 pages presented one after the other. They were not allowed to go back to previously viewed pages, but they were allowed to choose up to 15 pictures of possible culprits, which would be shown again later on a final screen. After viewing all 300 pictures, those previously chosen pictures were shown again, and participants could pick one final picture of a culprit or choose “culprit absent.” Following the mugbook they were asked how certain they were about their decision and about their strategy underlying their decision.

**FIGURE 2:**
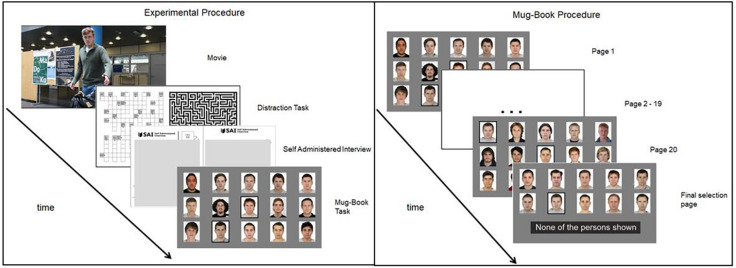
Experimental procedure (left panel): After a movie of a staged crime was shown, participants were given a distractor task for exactly 10 min. Then they were given a Self-Administered Interview (SAI), after which they were asked to complete the mugbook task. Mugbook procedure (right panel): Participants looked through 20 pages with each 15 pictures and were able to choose a person if they think they recognized the culprit. All people selected on the 20 pages were shown on the last page where participants could make a definitive selection or reject the mugbook search.

Finally, they were presented with an eight-person line-up, with the culprit included and they could again choose if the culprit was present (and indicate the person) or not. After testing, participants were fully debriefed. A visual depiction of the Experimental procedure can be seen in Figure 2.

#### SAI Statement Analysis

Statement analysis of the SAI was conducted by two independent coders who were blind to the outcome of the mugbook search. Statements were analyzed using a scoring template. The template was generated according to the information presented in the movie. Information about the culprit was collected concerning facial characteristics and additional characteristics. For each item every individual characteristic of this item was scored with two points if mentioned correctly. For example, a statement as “short, straight, brown hair” would have been given six points altogether (short = 2, straight = 2, and brown = 2). Two points were given for a correct item to also allow partly correct statements, which were given one point (e.g., if the participant would mention dark hair instead of brown hair). Incorrect statements about an item were given zero points.

Additionally, a category was created for statements that did not contain falsely reported additional features not previously accounted for in other items. If no false additional features were reported, participants received two points in this category. If only minor false additions were made (things that should not affect the recognition greatly; e.g., suspect wore a chain around the neck), one point was given and if major (things that affect the recognition more prominently; e.g., suspect wore a hat) or more than two minor false additions were stated, zero points were given. Items were included into categories, and points were added together accordingly. Finally, scores were made for features mentioned about clothing, general characteristics (e.g., height, age), external facial features (e.g., hair, head shape), internal facial features (e.g., eyes, nose), total facial features (internal and external facial features), and a score for the total number of features mentioned.

Since experiment 1 and 2 included different ratings strategies, we would like to give our reasoning for this decision. The different rating strategies included in experiment 1 vs. experiment 2 were based on the different cognitive mechanisms that we thought that participants would use when mentioning correct attributes. In experiment 1, the goal was to assess if the suspect would be included in the mugbook search, meaning that a correct answer even on the most general level would include the suspect. More precisely, the scientific interest was primarily in falsely remembered attributes where the suspect would be excluded from the lineup. Therefore as long as the answer was not wrong, the accuracy of the description is sufficient. In experiment 2, we wanted to assess how accurately the suspects were remembered, meaning we had to differentiate participants who give answers on a different level of accuracy. As the goal was to assess if a better accuracy of remembering would offer a better recognition, we had to differentiate e.g., “dark hair” from “dark brown hair,” whereby the later would be a better memory. However, both of these descriptions would lead to the inclusion of the suspect in the mugbook in experiment 1.

### Results

The results showed that 22.2% (*n* = 20) of participants who viewed a culprit present mugbook were able to identify the culprit correctly, that is, the culprit was chosen in the final decision page when all previously selected culprits were shown again. Also 50% (*n* = 14) of the people who viewed a culprit absent mugbook were able to reject the mugbook search reporting that the culprit was not present in the mugbook. Decisions where a culprit has been identified correctly in a culprit present mugbook and where a culprit absent mugbook was rejected were labeled “correct decisions” for later analysis. Incorrect decisions were on the one hand false person identifications, as in 37.8% (*n* = 34) of culprit present mugbooks and 50% (*n* = 14) of culprit absent mugbooks, and on the other hand no-present decision of a mugbook if the culprit was actually present, as made in 40% (*n* = 36) of the cases. A resume of these results can be found in Table 2. Demographic differences and differences in decision rates between the results for each culprit were marginal. However, it has to be mentioned that the proportion of female participants in the experiment was not equally distributed between both culprits. Yet, this did not affect the results as analyses were only made for culprits combined.

**TABLE 2:** Mugbook selection numbers for each culprit and in total.

	**Witness ID decision**		
**Culprit condition**	**ID of culprit**	**ID of innocent culprit**	**No ID**
**Culprit A**			
Present (*n* = 45)	24.4% (11)^a^	33.3% (15)^b^	42.2% (19)^b^
Absent (*n* = 14)	Cannot occur	64.3% (9)^b^	35.7% (5)^a^
**Culprit B**			
Present (*n* = 45)	20% (9)^a^	42.2% (19)^b^	37.8% (17)^b^
Absent (*n* = 14)	Cannot occur	35.7% (5)^b^	64.3% (9)^a^
**Total**			
Present (*n* = 90)	22.2% (20)^a^	37.8% (34)^b^	40% (36)^b^
Absent (*n* = 28)	Cannot occur	50% (14)^b^	50% (14)^a^

*Experiment 2 Numbers in parentheses are frequencies, all foils in the mugbook are considered innocent culprits. Total equals the numbers of Culprit A and Culprit B combined, *N* = 118, ID = Identification. Adapted from “Eyewitness identification research: Strengths and weaknesses of alternative methods” by Wells and Penrod (2011).*

*^*a*^Correct decisions concerning the mugbook task.*

*^*b*^Incorrect decisions concerning the mugbook task.*

Analysis of the statements concerning the agreement between the two coders on the total scores of the five categories (“clothes,” “general features,” “external facial features,” “internal facial features,” “total facial features,” and “total features”) were analyzed using a two-way-mixed, absolute agreement, average-measures ICC (Intra-class correlation). Excellent reliability measures were obtained (Koo and Li, 2016) for clothing (ICC = 0.980), total facial features (ICC = 0.969), external facial features (ICC = 0.970), internal facial features (ICC = 0.929), and total features (ICC = 0.967). General features (ICC = 0.869) showed a marginally lower, but still good, reliability (Koo and Li, 2016). A summary table of the ICC can be found in Table 3.

**TABLE 3:** Results of the ICC quantification for the statement analysis in study 2.

	***ICC***	**95% *CI***		***F* test With True Value 0**			
**Feature category**		***LL***	***UL***	***F***	***df*1**	***df*2**	***p***
Clothing	0.980	0.971	0.986	48.724	117	117	<0.001
General features	0.869	0.805	0.911	8.130	117	117	<0.001
Total facial features	0.969	0.955	0.978	31.860	117	117	<0.001
External facial features	0.970	0.956	0.979	32.871	117	117	<0.001
Internal facial features	0.929	0.898	0.951	14.008	117	117	<0.001
Total features	0.967	0.952	0.977	31.572	117	117	<0.001

*ICC measures conducted in SPSS Using Average-measure, Absolute-Agreement, 2-Way Mixed-Effects Model, *ICC* = Intra-class-correlation, *CI* = Confidence Interval, *LL* = Lower Limit, *UL* = Upper Limit. Adapted from “A Guideline of Selecting and Reporting Intraclass Correlation Coefficients for Reliability Research” by Koo and Li (2016).*

#### Culprit Description and Identification

**FIGURE 3:**
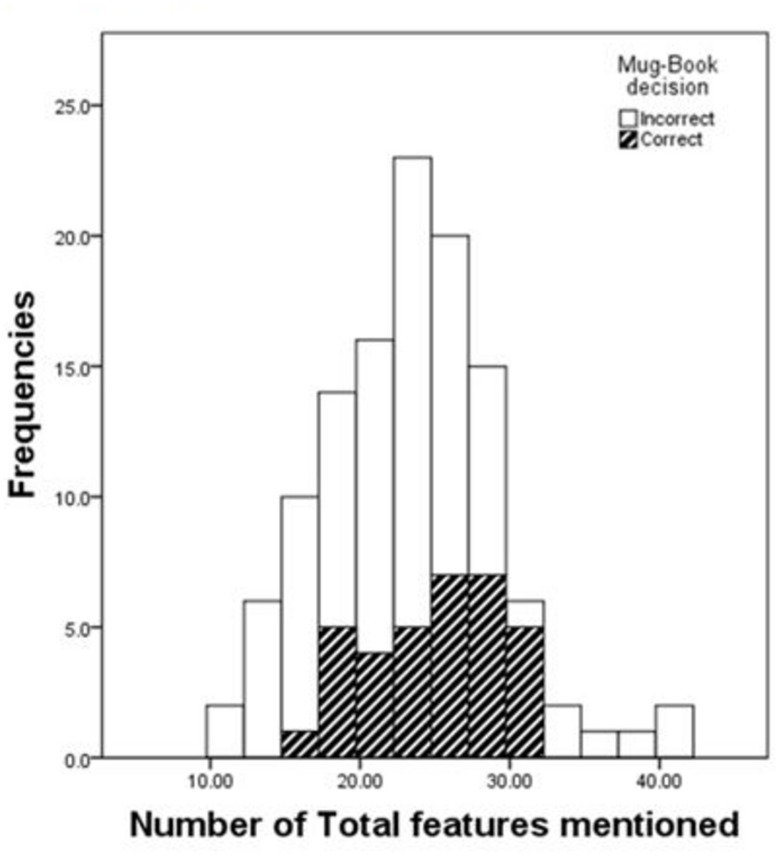
Distribution of the number of features mentioned for correct and incorrect mugbook decisions. Frequencies represent the number of witnesses that mentioned the number of features.

To calculate the relationship between written statements of a witness and the accuracy of the witness in the mugbook search, the total amount of features mentioned was analyzed for correct and incorrect mugbook searches as seen in Figure 3. According to the Kolmogorov-Smirnov test the distribution in the correct (*D* = 0.078, df = 84, *p* = 0.200) and the incorrect (*D* = 0.127, df = 34, *p* = 0.179) mugbook searches follows a normal distribution. Additionally, a non-significant Levene’s test (*F* = 1.642, df = 116, *p* = 0.203) shows homogeneity of variances between the samples. Therefore, a one-tailed independent *t*-test assuming equal variances was used to analyze differences in means between the groups.

Participants who identified the culprit correctly in the mugbook (*M* = 24.912, SD = 4.396, *n* = 34) compared to people who did not correctly identify the culprit in the mugbook (*M* = 22.661, SD = 6.160, *n* = 84), show a significantly higher average amount of correct statements about the culprit (*t*116 = 1.938, *p* = 0.028). An effect size of *d* = 0.394 according to Cohen (1992) represents a small to medium effect. Additionally, to analyze the relationship between total features mentioned and correctness in the mugbook search a point-biserial correlation was conducted. There was a one-tailed significant positive correlation between the number of features mentioned about the culprit in total and mugbook accuracy (*r* = 0.177, *p* = 0.028). A correlation of *r* = 0.177 shows a rather small effect (Cohen, 1992). These results show that people who were able to state more information about the culprit in the previous questionnaire also tend to be better in the recognition task later on.

Additionally, statements were analyzed concerning external and internal facial features. Descriptive statistics concerning the amount of external and internal facial features mentioned by participants can be seen in Table 4. As the distribution of Correct statements made about external (*D* = 0.100, df = 118, *p* = 0.006) and internal (*D* = 0.216, df = 118, *p* < 0.001) features did not follow a normal distribution according to the Kolmogorov-Smirnov test, a Wilcoxon Signed-Rank test was conducted. Participants stated significantly more information concerning external facial features compared to internal facial features (*z* = −9.155, *p* < 0.001). An effect size of *d* = 3.132 can be considered a large effect size.

**TABLE 4:** Descriptive statistics for external and internal facial features of culprits reported by participants.

**Features**	***Mean***	***SD***	***Median***	***Min***	***Max***
External	7.46	3.202	7	0.5	14
Internal	2.33	1.831	2	0	9.5

*Experiment 2, Descriptive statistics of external and internal facial features, *n* = 118, SD = Standard deviation, Min = minimal amount of points given for a statement, Max = maximal amount of points given for a statement.*

To test if the amount of correct statements about external facial features is related to the accuracy in a following mugbook task, the number of correct statements were analyzed depending on correctness of the later mugbook search. The number of correct statements made about external features did not follow a normal distribution, according to the Kolmogorov-Smirnov test (*D* = 0.116, df = 84, *p* = 0.007), for people who made an incorrect decision on the mugbook task. Therefore, a one-tailed Mann-Whitney-*U* test was conducted. Results show that people who were not correct in the mugbook task mentioned less correct statements about external features compared to those who were correct (*U* = 1065.5, *p* = 0.015). Effect size being *d* = 0.406, meaning this is a small to medium effect. A point-biserial correlation of external facial features mentioned and identification accuracy supported these results showing that there was a significant positive correlation between the two variables (*r* = 0.198, *p* = 0.016, one-sided significant), meaning that correct identifications were associated with more correct statements to external features. Yet, a correlation of *r* = 0.198 is a rather small effect.

#### Predictive Witness Confidence

Total distributions of predictive confidence levels can be seen in Table 5. Overall, 25.3% of participants (*n* = 30) rated their confidence on the lower half of the scale, while 74.7% (*n* = 88) of the participants were on the upper half of the scale, meaning they were rather confident. To analyze the relationship between predictive confidence and the accuracy, a Spearman’s rank-order correlation was conducted. Results for this analysis were not significant (*r* = −0.042, *p* = 0.326), indicating that predictive confidence and mugbook accuracy are not related.

**TABLE 5:** Identification accuracy according to the predictive confidence level.

**Mugbook choice**	**Predictive Confidence**					
	**1**	**2**	**3**	**4**	**5**	**6**
Accurate	0 (0%)	1 (11.1%)	8 (40%)	15 (34.9%)	6 (17.1%)	4 (40%)
Inaccurate	1 (100%)	8 (88.9%)	12 (60%)	28 (65.1%)	29 (82.9%)	6 (60%)
Total Number	1	9	20	43	35	10

*Experiment 2, Numbers are frequencies, percentage (%) in parentheses shows the amount of choices per confidence level. Confidence ratings resemble the choices participants made on the Likert Scale, with 1 being the least certain and 6 being the most certain.*

### Discussion

The goal of experiment 2 was to assess if the quality of the culprit description given by the witness is positively correlated with the ability to correctly identify the culprit in a subsequent mugbook search. Here, the participants who mentioned more features of a culprit in total tended to be more accurate in their decision when trying to identify the culprit in a mugbook search. These results are in line with early assumptions made by the United States and German supreme court, which assumed that the quality of a person description was an indicator in the evaluation of the accuracy of person identifications in criminal trials (Sporer and Cutler, 2003).

However, correct statements and descriptions alone should not be considered as the sole indicator for identification accuracy. First, the effect size in our study was only small to medium. Second, if results are considered individually, the six participants who mentioned the most features all made an incorrect mugbook decision. Third, in this experiment only correct statements were counted and not the total amount of statements. In real life, the accuracy of statements cannot be verified prior to the mugbook search and sometimes even after the search (Fahsing et al., 2004). Yet, our results can nonetheless be seen as optimistic as the ability to give a correct verbal description of a culprit seems to be related to the ability to correctly identify the culprit in a mugbook task.

To analyze which specific facial features were more important for the identification task and the witness, two separate hypotheses were tested. First, witnesses were expected to describe significantly more external than internal features, and our participants indeed mentioned a larger number of external compared to internal facial features. This is in line with previous research showing that internal features were rarely mentioned in eyewitness descriptions (Lindsay et al., 1994a; Van Koppen and Lochun, 1997). Second, a positive correlation between the number of correctly remembered external facial features with identification accuracy was expected. Participants who either correctly rejected a culprit absent in the mugbook or who identified the culprit in a culprit present mugbook, tended to give more external facial descriptors compared to those who made a false identification or missed the culprit. This supports the assumption that external features play an important role in the recognition of unfamiliar faces (Ellis et al., 1979; Davis and Loftus, 2018). However, a problem with these features is that while they might be easily expressible, they might not always be useful to differentiate between faces (Meissner et al., 2007). External features such as hair color or length may be changed rather easily, or they can be easily disguised, e.g., by wearing a hat. These simple changes in the external features can lead to impaired recognition accuracies (Chan and Ryan, 2012). Especially in mugbook searches where pictures might be rather old, there is a high probability that some certain external face features might have changed in potential culprits.

The final goal of experiment 2 was to assess if predictive confidence will show a positive correlation with actual performance in mugbook searches. We, however, did not find evidence for this proposed association here. There was no relation between predictive confidence and identification accuracy in the mugbook search, even though they were made after a brief delay and not immediately after viewing the culprit. These findings differ from line-up studies, which did find a relation between predictive confidence and identification accuracy, when confidence ratings were made after a brief delay (Nelson and Dunlosky, 1991; Nguyen et al., 2018). One reason for this might be the way how witnesses form their accuracy assumption concerning a later identification task. According to Leippe et al. (2009), the sources that witnesses use for their estimates are self-credibility, intrinsic sources, and extrinsic cues. While intrinsic sources and self-credibility are person-dependent (Leippe et al., 2009) and therefore do not differ from mugbook to line-up searches, extrinsic cues also include witnesses’ beliefs about the identification task. For the witness, it might be difficult to estimate how challenging a mugbook task is, and therefore, they cannot properly estimate how accurate they will be in a following mugbook search.

## General Discussion

The aim of the experiments reported here was to analyze if there were any differences between correct and incorrect mugbook searches when looking at prior culprit descriptions and information in the preparatory phase. Especially we wanted to assess if a positive outcome can be predicted after an initial statement and description about the culprit is given by the witness.

Experiment 1 showed that there is a high risk that the real culprit is not included in the mugbook if a matching approach (i.e., when pictures are selected according to the description) is chosen, and feature criterions are applied too strictly. Yet some matching has to be done due to the large number of pictures in certain population settings (Lindsay et al., 2017) but also because a greater mugbook size decreases the chance of correct identifications (McAllister et al., 2003; McAllister and Lindsay, 2007; Kalmet, 2016). The challenge, therefore, is to analyze which culprit description items should be considered or disregarded in the preparation for the mugbook search. According to our findings, age within a range of ±5 years seems to be an adequate criterion to down-scale the mugbook, but culprit height was not well estimated and might be more problematic. However, results from previous studies show that height estimates were more accurate compared to age estimates (Fahsing et al., 2004). It seems that the decision, which items should be used to minimize the mugbook size, cannot be answered in general but is more a case-to-case question. Yet how this decision is made is challenged by the fact that results in our experiment did not show a correlation between subjective memory strength estimations and accuracy in descriptions. This suggests that attributes, which are remembered with higher certainty, should not automatically be given a higher value.

This highlights the notion that information that could affect identification accuracy should be carefully taken into account. First, persons are better at estimating someone of their own age group (Voelkle et al., 2012). If the witness estimates the culprit to be in his/her age group, accuracy in statements is more probable and the attributes can be given more attention. Second, for height estimates, people tend to use eye height scaling (Twedt et al., 2012), meaning that persons showed better accuracy in height estimates when the height of the culprit was not much different from the eye-height of the participant (Twedt et al., 2015). This means that if the eye-height of the witness during the crime was about the same height as the culprit during the crime, a smaller margin of error can be chosen when height estimates are analyzed. Also, height estimations seem more accurate if they are done in the same body position as at the time of encoding the height (Twedt et al., 2012). The results of Twedt and colleagues also show that ideally a matching approach (presentation of the pictures are limited to those that match the description) is only used if really necessary; it seems overall better to use a sequencing approach (pictures are sorted that pictures which better match the description are presented earlier) for as many attributes as possible. Especially, as hair color for example is easily changeable and eye color was not remembered correctly by many participants, it seems suboptimal to eliminate pictures according to eye or hair color. The use of these easily changeable and not often accurately remembered attributes should therefore be limited to a use in a sorting procedure.

Interestingly in Experiment 1, there was no correlation between self-estimation of how well a witness could remember the culprit and the accuracy of the person description. In Experiment 2, however, self-assessment of memory abilities for remembering culprit features showed a significant positive correlation to actual attributes remembered about the culprit, though this correlation was limited to the categories “total features” (*r* = 0.293, *p* = 0.001), “facial features” (*r* = 0.199, *p* = 0.031), “external features” (*r* = 0.195, *p* = 0.035), and “clothes” (*r* = 0.242, *p* = 0.008). On the other hand, general features, which also include age, height, and ethnicity of the culprit, did not show a relationship to the witnesses’ memory estimations. If witnesses were able to analyze how well they remembered features that are visible but not those that have to be estimated prior to memorizing them, we might conclude that people did not forget these attributes but instead that they had difficulties with making accurate estimates from the beginning.

This seems reasonable as there might be several factors affecting estimations about other person’s attributes. For example, the witness’s height estimation might be influenced by the participants’ own knowledge or assumptions of the average population norm (Meissner et al., 2007). Furthermore, body scaling or eye height scaling is used to making estimations (Twedt et al., 2012, 2015), such that persons use their own eye-height to estimate these features in other persons. In our experiments, however, all participants were seated when they viewed the culprit, which did not allow them to use their own eye height to estimate the height of the culprit.

In experiment 2, an experimentally simulated mugbook search revealed that only 30 of 90 participants selected the culprit, and only 20 participants correctly identified the culprit in the final decision. This might seem to be a satisfactory identification rate, as 300 pictures had to be viewed, and the possibility of finding the right person by chance is 1–299. However, a critical notion concerns the fact that from 118 total mugbook searches, 48 wrong identifications were made. We have to note that the calculation of a diagnostic ratio was not performed here due to the fact that in mugbook searches, the difference between misses and false alarms is not as clear as in line-ups. In line-ups an innocent person is usually presented in the culprit absent line-up condition, as compared to real line-ups, where there is also one known culprit placed among innocent foils (Lindsay et al., 2017). Calculations in this case are usually made by considering foil identification in culprit present conditions simply as misses and false alarms as identification of the innocent culprit (Wells and Penrod, 2011). This allows a clear distinction between false alarms and misses. In mugbook searches. However, the culprit is not known and every foil has to be considered to be an innocent person. In this case, it is hard to decide if an innocent person that is wrongly identified in a culprit-present mugbook should be classified as a “false alarm” or as a “miss”? For this reason, no diagnostic ratio was created in our study. Yet, the high number of false identifications supports the assumption that mugbook searches should not be used as an identification tool but rather as an investigative tool (Lindsay et al., 1994b). A correct identification in the mugbook is thus not proof that the culprit has committed the crime but rather that this person should be further investigated.

The question remains why such a large number of false identifications were found in our study. One possible reason is that due to the design of the mugbook search, people are likely to use a relative judgment, where they choose the person who looks the most like the culprit, and they do not try to recognize the culprit specifically (Lindsay and Bellinger, 1999). To increase the chance of finding the culprit amongst all of the pictures, witnesses were allowed to choose more than one culprit in a first identification attempt. The grouped mugbook procedure should prevent the use of relative judgment (McAllister et al., 2008). However, once participants are allowed to choose more than one culprit, they could be tempted to select different pictures of people who look similar, eliminating the advantages of the grouped procedure. Pictures that were chosen initially were later presented simultaneously on the last page. It is possible that the number of false identifications could be reduced by presenting the last pictures sequentially. Sequential presentations generally seem to be superior for line-up identification and reduce the amount of false identifications (Wells and Lindsay, 1985). Nevertheless, as the process of a mugbook search should be considered rather as an investigative tool instead of an identification process (Lindsay et al., 1994b), it seems reasonable to allow a slightly higher false identification rate if it helps to increase correct identifications simultaneously.

One of the main motivations for our experiment was to assess if there are certain indicators that can be used to estimate if a mugbook search is advised or not. Here, accuracy in a mugbook search was positively correlated with the accuracy of a verbal statement about the culprit and the amount of external facial features correctly mentioned. However, estimation of the witnesses’ ability to identify the perpetrator in the mugbook task (predictive confidence) did not prove to be a good indicator for how accurate the witness is in a later mugbook search.

### Limitations and Future Research

There are certain aspects that could not be addressed in our experiments. Concerning experiment 1, findings can only be generalized for culprits who present the same attributes as those used in this sample. Also, ethnicity of our “culprits” was limited to western Europeans, such that recognition rates for culprits from other ethnicities are difficult to predict. While the participant sample in experiment 2 seemed to be more diverse overall, college students were represented above average. This might lead to the fact that eyewitness abilities are overestimated, as college students tend to be more accurate as eyewitnesses (Wells et al., 2006), and furthermore the size of the mugbook might be drastically bigger in large cities leading to lower results in real life mugbook searches (Lindsay et al., 2017). Second, there are numerous estimator variables that may influence recognition, such as racial bias, stress, weapon focus, exposure duration, disguise, and presentation delay (Wells et al., 2006).

A third limitation of our study is that the culprits did not significantly change the features of their face between movie and picture. In real-life mugbooks, it is possible that certain external facial features, such as hair color or length, may have changed drastically, especially if the culprit was registered for a previous offense long ago. The same question can be raised if the culprit has a very distinctive internal facial feature such as a very recognizable nose or a birthmark. In that case, it might be possible that mentioning of this internal feature will show a better correlation compared to external features.

Fourth, the Self Administrated Interview offers a tool to get as much information about the crime witnessed (Gabbert et al., 2009). While this tool offers the possibility to test the participants in a standardized way and minimizing any possible effect the experimenter would have when asking questions, it might not obtain all relevant information. Although the SAI uses techniques from the Cognitive interview to generate an elaborate statement about the crime (Gabbert et al., 2009), being able to personally ask questions (especially follow-up questions) about the crime by the investigator might reveal even more detailed statements and descriptions of any relevant culprit feature.

## Conclusion

Our data provide valuable insights in the accuracy of person descriptions in the preparation for and for performing of actual mugbook searches. Experiment 1 showed that the use of too many culprit features to down-scale the mugbook would lead to a high chance of the culprit being eliminated from the mugbook. Since it is generally important to limit the number of mugshots, attributes should not be chosen due to subjective certainty of the witness about his own memory accuracy but rather based on “objective” assessments of the witness’ accuracy as documented in other statements made by the witness. For uncertain culprit attributes, a sequencing approach should be prioritized. Experiment 2 showed that witness’ statements about the culprit could be used to assess the probability with which the witness can identify the culprit in a later mugbook search. A more elaborate witness’ statement increases the probability that the culprit will be identified. However, witnesses’ own estimations about his/her accuracy in a following mugbook search seems to have no major relevance.

Taken together, the results of our experiments allow a few recommendations about when a mugbook search might or might not be advisable. First, the witnesses’ predictive confidence should not be used as a major indicator. Predictive confidence did not show any correlation with the actual identification accuracy. Second, the witness’ statements about the culprit can be valuable information if the witness will be able to identify a possible culprit. As there is no absolute indicator about the success in the mugbook search, witnesses who are only able to describe very few details about facial features will probably perform worse in the subsequent mugbook search. Third, there are no absolute indicators that allow prediction on the outcome of a mugbook search. For example, witnesses who make very elaborate statements in the description phase mentioning a multitude of facial descriptors and having promising estimator variable features, can still make a false positive identification. Fourth, our study shows nevertheless that there are certain circumstances that make a positive outcome of the mugbook search more likely. Specifically, a mugbook search is more advisable if the witness is able to give an elaborated and convincing description of the potential culprit.

## Data Availability Statement

The raw data supporting the conclusions of this article will be made available by the authors, without undue reservation.

## Ethics Statement

Ethical review and approval was not required for the study on human participants in accordance with the local legislation and institutional requirements. The patients/participants provided their written informed consent to participate in this study. Written informed consent was obtained from the individual(s) for the publication of any potentially identifiable images or data included in this article.

## Author Contributions

Both authors listed have made a substantial, direct and intellectual contribution to the work, and approved it for publication.

## Conflict of Interest

The authors declare that the research was conducted in the absence of any commercial or financial relationships that could be construed as a potential conflict of interest.

## Publisher’s Note

All claims expressed in this article are solely those of the authors and do not necessarily represent those of their affiliated organizations, or those of the publisher, the editors and the reviewers. Any product that may be evaluated in this article, or claim that may be made by its manufacturer, is not guaranteed or endorsed by the publisher.
